# Emerging beneficial roles of sirtuins in heart failure

**DOI:** 10.1007/s00395-012-0273-5

**Published:** 2012-05-24

**Authors:** Masaya Tanno, Atsushi Kuno, Yoshiyuki Horio, Tetsuji Miura

**Affiliations:** 1 Second Department of Internal Medicine, Sapporo Medical University, S1 W16, Chuo-ku, Sapporo, 060-8556 Japan; 2 Department of Pharmacology, Sapporo Medical University, Sapporo, 060-8556 Japan

**Keywords:** SIRT1, SIRT3, Heart failure, Mitochondria, Metabolism, Oxidative stress

## Abstract

Sirtuins are a highly conserved family of histone/protein deacetylases whose activity can prolong the lifespan of model organisms such as yeast, worms and flies. In mammalian cells, seven sirtuins (SIRT1–7) modulate distinct metabolic and stress-response pathways, SIRT1 and SIRT3 having been most extensively investigated in the cardiovascular system. SIRT1 and SIRT3 are mainly located in the nuclei and mitochondria, respectively. They participate in biological functions related to development of heart failure, including regulation of energy production, oxidative stress, intracellular signaling, angiogenesis, autophagy and cell death/survival. Emerging evidence indicates that the two sirtuins play protective roles in failing hearts. Here, we summarize current knowledge of sirtuin functions in the heart and discuss its translation into therapy for heart failure.

## Introduction

Heart failure is a disease with multifactorial causes. Failing hearts present complex phenotypes, including myocyte loss, increased fibrosis, diminished response to stresses, loss of myocardial energetic reserve and reduced myocardial contractility. Accumulating evidence indicates that epigenetic modification represents a molecular substrate for cellular stresses, either suppressing or promoting disease initiation [71]. In particular, lysine residue acetylation is one of the important posttranslational modifications to regulate the function of proteins. Sirtuins mediate this posttranslational modification by coupling lysine deacetylation to NAD^+^ hydrolysis [116]. The dependence of sirtuin activity on NAD^+^ suggests that their enzymatic activity is directly linked to the energy and redox status of the cell via the NAD^+^/NADH ratio. Among the seven mammalian sirtuins, SIRT1 and SIRT3 have been most intensively investigated. Interestingly, SIRT1 and SIRT3 favorably modify cellular functions that may underlie the above-mentioned heart failure phenotypes. These sirtuins are crucially involved in regulation of cardiomyocyte energy metabolism, production of reactive oxygen species and signaling relevant to cell death/survival. Biological functions of SIRT1/SIRT3 are described in this review, mainly from the viewpoint of translation into therapy for heart failure.

## SIRT1 and SIRT3

**Fig. 1:**
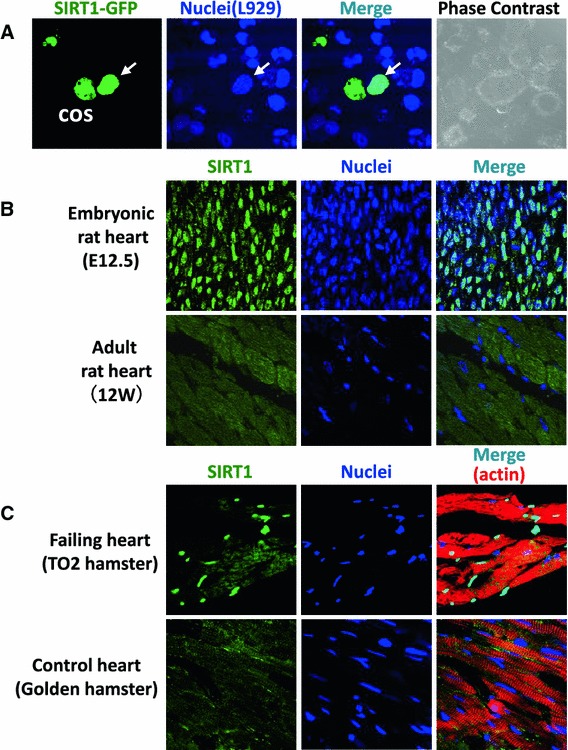
Nucleo-cytoplasmic shuttling of SIRT1 and its differential distribution. **a** L929 cells were pre-stained with Hoechst 33342 and then fused with COS7 cells transfected with SIRT1-EGFP (heterokaryon). SIRT1-EGFP was detected not only in COS7 nuclei but also in L929 nuclei (*arrows*), indicating that SIRT1-EGFP reached the L929 nucleus via the heterokaryon cytoplasm. SIRT1-EGFP, Hoechst 33342 nuclear staining, merged images, and phase-contrast images are shown. **b** Immunofluorescence staining reveals that embryonic (E12.5) mouse hearts express SIRT1 predominantly in the nuclei, whereas SIRT1 was diffusely distributed in the cytoplasm in adult (3-month-old) mouse hearts. **c** The expression levels of nuclear SIRT1 in the cardiomyocytes were much higher in severely failing hearts from TO-2 hamsters than in non-failing control hearts. Produced from ref [118] with permission

Mammals express seven sirtuins, SIRT1–7, of which the molecular weights range from 34 to 62 kDa [78]. All have an NAD^+^-dependent catalytic core domain that may act as an NAD^+^-dependent deacetylase and/or mono-ADP-ribosyl transferase. Additional N-terminal and C-terminal sequences of variable lengths flank this core domain [78]. SIRT1 is highly expressed in mammalian hearts and regulates a wide array of cellular processes by deacetylating histones and a number of non-histone proteins [38]. SIRT1 has been demonstrated to be localized predominantly in the nuclei or cytoplasm depending on the cell type. SIRT1 shuttles between the two cellular compartments in response to cellular stress in C2C12 cells and cardiomyocytes [117, 118] (Fig. 1), and during differentiation in neural precursor cells [50]. The nucleo-cytoplasmic shuttling is regulated by nuclear localization signals and nuclear export signals in the amino acid sequences of SIRT1. PI3K/Akt- and JNK1-mediated phosphorylation of SIRT1 induces its nuclear translocation [83, 118]. Nuclear localization of SIRT1 seems to be necessary for its protective function in cardiomyocytes [117, 118], whereas the biological significance of cytoplasmic SIRT1 remains to be determined.

SIRT3 has been reported to be a major mitochondrial deacetylase, although SIRT4 and SIRT5 also reside in the mitochondria [69]. The full-length human SIRT3 is a 44-kDa protein with an N-terminal mitochondrial localization sequence [91]. Following import to mitochondria, 142 amino acids from the N-terminus of full-length SIRT3 are cleaved to generate an active 28-kDa short form [91]. Sundaresan et al. [115] reported that the endogenous long-form SIRT3 was localized in the mitochondria, cytoplasm and nuclei, whereas the short form of SIRT3 was detected only in the mitochondria in hearts. Although SIRT3 has been believed to be a “mitochondrial sirtuin”, full-length SIRT3 in the nucleus is also enzymatically active as indicated by its ability to deacetylate H3 and H4 [101].

As opposed to these findings, a recent study demonstrated that the endogenous SIRT3 was only evident in mitochondria and not in the nuclear compartment in H9c2 cells and MEF cells, whereas overexpressed FLAG-tagged SIRT3 constructs were detected in sucrose gradient purified nucleus [12]. These data raise interesting questions as to whether biological functions of SIRT3 are actually operational in different subcellular compartment, or whether the robust overexpression of the protein may evoke artifactual localization.

## Regulation of mitochondrial function and energy utilization by sirtuins

The role of energy deficits in the development and progression of heart failure is well established as described below. In physiological conditions, hearts predominantly use free fatty acid (FFA) for ATP production (i.e., approximately 70 % of total ATP) [4]. In the early stage of heart failure, the heart switches the substrate to glucose, which produces more ATP per molecule of oxygen consumed than FFA, at the expense of low energy yield compared with the yield in FFA oxidation. In advanced heart failure, however, insulin resistance develops in the myocardium and glucose utilization declines, limiting ATP production [128]. Indeed, high-energy phosphate levels have been shown to be correlated directly with survival in cardiomyopathy patients [85]. A number of therapies for heart failure by modulation of myocardial metabolism have been tested and some of them showed promising results [8]. Administration of glucagon-like peptide 1 in a canine pacing-induced cardiomyopathy resulted in increased GLUT1 expression [17] and significantly improved cardiac function [86]. Dichloroacetate, an inhibitor of pyruvate dehydrogenase kinase, augmented glucose and pyruvate metabolism, leading to improved ejection fraction in patients with heart failure [16]. Etomoxir inhibits mitochondrial carnitine palmitoyltransferase-1 (CPT-1) and subsequently suppresses long-chain FFA oxidation [70]. The reduction of FFA oxidation by etomoxir was associated with pyruvate dehydrogenase and phosphofructokinase, leading to enhanced glycolysis and glucose oxidation [104]. A small pilot study revealed that administration of etomoxir significantly improved left ventricular ejection fraction (LVEF) and cardiac output during exercise in patients with ischemic and dilated cardiomyopathy [103]. Perhexiline, a potent inhibitor of CPT-1 and CPT-2, also improved LVEF, symptoms of heart failure, MVO_2max_ and skeletal muscle energetics in a small clinical study [63].

**Fig. 2:**
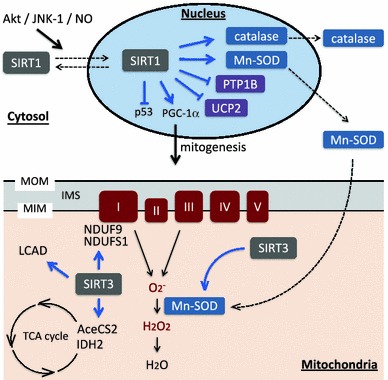
Diverse roles of SIRT1 and SIRT3 in regulation of ATP production and oxidative stress. SIRT1 shuttles between the nucleus and cytosol. Nuclear localizing signals and nuclear export signals are necessary for transport through the nuclear membrane, and the shuttling is regulated by Akt, JNK-1 and/or NO. In the nucleus, SIRT1 up-regulates ROS scavengers, Mn-SOD and catalase, and down-regulates UCP2 and PTP1B. SIRT1 inactivates P53 and activates PGC-1α by deacetylating the lysine residues. Nuclear-encoded Mn-SOD translocates into the mitochondria and catabolizes highly toxic O_2_^−^ into less toxic H_2_O_2_, which in turn is detoxified to water. In the mitochondria, SIRT3 deacetylates and activates TCA cycle enzymes (AceCS2 and IDH2), ETC enzymes (NDUF9 and NDUFS1) and fatty acid oxidation enzyme (LCAD). SIRT3 also enhances activity of MnSOD by deacetylation. *MOM* mitochondrial outer membrane, *MIM* mitochondrial inner membrane, *IMS* inter-membrane space, *PTP1B* protein tyrosine phosphatase 1B, *UCP2* uncoupling protein 2, *AceCS2* acetyl-CoA synthetase 2, *LCAD* long-chain acyl co-enzyme A dehydrogenase, *IDH2* isocitrate dehydrogenase 2

In this context, mitochondrial dysfunction may be one of the important factors involved in deterioration of ventricular contractile functions, because mitochondria are integrally involved in energy production and metabolism in the heart [77]. SIRT1 and SIRT3 regulate mitochondrial functions by deacetylation of nuclear proteins and mitochondrial proteins, respectively [73, 78], as described in detail in the following “Increased energy production by SIRT1: indirect modulation of mitochondrial function” and “Evidences arguing against the benefits afforded by SIRT1 in transgenic mice”. A recent proteomic survey revealed that most proteins with acetylation residues were localized in the mitochondria and/or associated with energy metabolism [57], arguing for involvement of sirtuins. Functional properties and expression of mitochondrial proteins is controlled at both the mitochondrial and nuclear genome levels. Among approximately 1,500 proteins in the mitochondrial proteome, only 13 are expressed by the mitochondrial genome; the other mitochondrial proteins are encoded in the nuclei, synthesized in the cytosol as preproteins that include amino-terminus mitochondrial localization signal (MLS) and then transported into the mitochondria [51]. SIRT1 and SIRT3 play diverse roles in regulation of energy production and oxidative stress as shown in Fig. 2.

### Increased energy production by SIRT1: indirect modulation of mitochondrial function

SIRT1 suppresses expression of uncoupling protein 2 (UCP2), a mitochondrial inner membrane protein, in mouse pancreatic cells [20]. Since UCP2 functions to uncouple oxygen consumption from ATP production [53], suppression of UCP2 expression by SIRT1 results in increased mitochondrial ATP production [18, 31]. In addition, SIRT1 induces a substrate shift for energy production. SIRT1 interacts with and deacecylates PPAR gamma coactivator-1α (PGC-1α), a master switch of mitochondrial biogenesis, and induces gluconeogenic genes, resulting in increased glucose output in mouse liver and skeletal muscle [64, 97]. PPAR gamma coactivator-1α also regulates fuel utilization in muscle cells by increasing fatty acid oxidation and shutting down glucose oxidation [35]. The level of PGC-1α protein in failing human hearts was lower by approximately 30 % than that in non-failing control hearts [34], and decreased PGC-1α mRNA levels were associated with impairment of mitochondrial biogenesis and function in rodent models of heart failure [7, 126]. These findings indicate that insufficient activity of PGC-1α may lead to mitochondrial dysfunction and heart failure. Collectively, activation of PGC-1α by SIRT1 and enhanced mitogenesis may restore energy metabolism in the failing myocardium and ameliorate heart failure. Consistent with this notion, resveratrol, a SIRT1 activator, preserved mitochondrial mass and biogenesis and suppressed cardiac dysfunction in Dahl salt-sensitive rat fed with a high-salt diet, a model of hypertensive heart failure [96].

The improved insulin sensitivity by SIRT1 is also a potential mechanism by which SIRT1 preserves contractile function in failing hearts. It has been demonstrated that resveratrol, an SIRT1 activator, improves insulin sensitivity in diet-induced obesity in mice [13, 60]. Sun et al. [113] found that SIRT1 repressed protein phosphatase 1B (PTP1B) and thereby increased the level of insulin receptor phosphorylation, improving insulin sensitivity both in C2C12 myotubes and in high fat-fed mice. Resveratrol ameliorated pathological histology, such as vacuolization, degeneration and inflammation, in the hearts of high fat-fed mice with impaired insulin sensitivity [13]. PPAR gamma coactivator-1α has been shown to be decreased in aging murine hearts [121], which may be due, at least in part, to decreased SIRT1 expression [98]. The decrease in PGC-1α protein in hearts may be responsible for the predisposition of aging hearts to heart failure. Interestingly, a recent study demonstrated that SIRT1 and PGC-1α are localized in mitochondria and form a multiprotein complex with mitochondrial transcription factor A in mouse liver and HeLa cells [5], suggesting their involvement in mitochondrial biogenesis not only by regulation of nuclear-encoded proteins, but also by direct control of mitochondrial gene expression.

### Evidences arguing against the benefits afforded by SIRT1 in transgenic mice

In contrast to generally favorable effects of pharmacological activation of SIRT1 on the heart, results from SIRT1-overexpressing animals have been somewhat contradictory. Alcendor et al. [2] demonstrated that moderate overexpression of Sirt1 up to 7.5-fold attenuated age-dependent cardiac dysfunction and oxidative stress-induced apoptosis in mouse hearts, whereas a higher level (12.5-fold) of overexpression of Sirt1 increased apoptosis and hypertrophy and decreased cardiac function. Similarly, Kawashima et al. [54] demonstrated that constitutive cardiac-specific overexpression of SIRT1 at a high level (20-fold) caused dilated cardiomyopathy and that moderate (6.8-fold) overexpression of SIRT1 impaired cardiac diastolic function. Furthermore, fatty acid uptake was decreased, degenerated mitochondria increased and the expression of genes relevant to mitochondrial function decreased, in proportion to SIRT1 gene dosage. Transgenic mice with an even lower level of SIRT1 overexpression (3.2-fold) developed cardiac dysfunction upon pressure overload, although their basal cardiac function was preserved. Oka et al. [88] also provided evidence that overexpression of SIRT1 may deteriorate mitochondrial function and exacerbate cardiac dysfunction by suppressing expression of genes regulated by estrogen-related receptors in cardiomyocytes.

The reason for the discrepancy concerning the effects on ventricular functions between pharmacological activation and overexpression of SIRT1 is unclear, but there are some plausible explanations. First, gene dosage may have been too high even with low-level overexpression. Although a larger amount of SIRT1 is expected to produce a higher level of deacetylase activity, too much SIRT1 protein may cancel the protective effect through non-specific deacetylation and/or deacetylase-independent detrimental effects, e.g., interaction with other proteins and un-physiological subcellular distribution. Second, consequences of constitutive activation of SIRT1 may be different from those of temporary or intermittent activation. In agreement with this notion, transient transfection of SIRT1 deacetylated PGC-1α and increased fatty acid oxidation in a skeletal myocyte cell line [35], whereas stable transfection with SIRT1 impaired cellular respiration in PC12 cells [84]. Third, beneficial effects afforded by SIRT1 might not be applicable to myocardial damage induced by certain stressors. Kawashima et al. [54] and Oka et al. [88] employed severe pressure overload by transverse aortic constriction to induce heart failure. They demonstrated that overexpression of SIRT1 further promoted cardiac dysfunction, whereas Alcendor showed that moderate overexpression of SIRT1 rendered hearts resistant to oxidative stress-induced damage by paraquat.

### Increased energy production by SIRT3: direct modulation of ATP-producing machinery

SIRT3 appears to play a role in cellular energy production at multiple steps, i.e., up-regulation of fatty acid oxidation, supply of intermediates for the TCA cycle and activation of the electron transport chain (ETC). SIRT3 interacts with acetyl-CoA synthetase 2 (AceCS2), a cardiac-enriched mitochondrial enzyme that promotes entry of acetate into the tricarboxylic acid cycle in the form of acetyl-CoA, in Cos-7 cells. Sirt3 deacetylates lysine 642 of AceCS2 and activates its enzymatic activity [42]. SIRT3 also targets long-chain acyl co-enzyme A dehydrogenase (LCAD). Mass spectrometry of mitochondrial proteins in liver extracts from SIRT3^−/−^ mice has shown that LCAD is hyperacetylated at lysine 42. Hyperacetylation of LCAD reduces its enzymatic activity, suggesting that deacetylation of LCAD-K42 by SIRT3 increases fatty acid oxidation output [49]. Furthermore, exogenously transfected myc-tagged SIRT3 physically interacts with NDUF9, a subunit of complex I in the mitochondrial ETC, and deacetylates and activates the enzyme, thus augmenting ATP production in SIRT3^−/−^ mouse embryonic fibroblasts (MEFs) [1]. A recent study demonstrated that activities of ETC complexes I, III and IV, were decreased by approximately 30, 70 and 60 %, respectively, in livers of *Sirt3* ^−/−^ mice fed a high-fat diet compared with the activities in wild-type animals on the same diet [55].

Most of the above-mentioned findings were demonstrated in the liver tissue. However, it is likely that SIRT3 is also a major regulator of ATP synthesis in cardiac mitochondria, as indicated by the following evidences. First, prolonged caloric restriction, an intervention known to activate SIRT1/SIRT3 and extend lifespan in several species, including rhesus monkeys [30, 122], decreased the amount of acetylated forms of NDUFS1 in complex I and Rieske subunit of cytochrome bc1 in complex III in cardiomyocytes [107]. Second, mice lacking SIRT3, which exhibit a striking hyperacetylation of mitochondrial proteins [69], showed 50 % lower basal levels of ATP in the heart [1].

Unexpectedly, the SIRT3^−/−^ mice showed normal systolic function despite the substantial reduction of ATP [114]. This apparent contradiction may be explained by at least a few possibilities. First, diastolic function may be more susceptible to the decline in the level of myocardial ATP than systolic function. Indeed, there was a significant correlation between the ATP level and pulmonary capillary wedge pressure, but not ejection fractions in human hearts [111]. Second, ATP levels measured in whole heart may reflect neither ATP levels in close proximity to cytosolic ATPases, including myosin ATPase and SERCA, nor rate of ATP utilization by these enzymes. In this regard, the level of phosphocreatine and creatinine kinase activity, molecules that regulate energy transport and utilization, may be more closely associated with the contractile function than the level of ATP [59, 85]. In contrast, Hirschey et al. [49] demonstrated that ATP levels in the liver were normal at baseline in SIRT3^−/−^ mice, though 24-h fasting resulted in significantly lower hepatic ATP levels in SIRT3^−/−^ mice than in wild-type mice. These findings indicate that the cellular energy production/utilization is maintained by redundant mechanisms. They may partly compensate for each other when one is ablated, but the compensation may fail when the energy demand increases under stress conditions.

## Regulation of ROS by sirtuins

Reactive oxygen species (ROS) damage macromolecules within and outside the mitochondria. The mitochondrial ETC is the main source of ROS in most cells [11]. Hearts consume large amounts of O_2_ and yield high levels of ROS in the mitochondria. In addition, various extracellular factors, such as angiotensin II and tumor necrosis factor-α, induce ROS formation and promote cardiomyocyte death together with the mitochondrial ROS [36]. Among the many anti-oxidant defense systems, mitochondrial manganese superoxide dismutase (Mn-SOD) plays a pivotal role in the detoxification of ROS. Mice deficient in Mn-SOD have an enlarged heart with endocardial fibrosis and die within the first 10 days of life [66]. Furthermore, long-term reduction of Mn-SOD activity by heterozygous deletion of the Mn-SOD gene impairs left ventricular functions: MnSOD^+/−^ mice displayed a decrease in ejection fraction and developed cardiac hypertrophy with fibrosis and necrosis [68]. In the clinical arena, patients with hemochromatosis, in which iron overload induces robust oxidative stress, have a significantly higher prevalence of cardiomyopathy if MnSOD activity is reduced by a mutation in the Mn-SOD gene [123].

Both SIRT1 and SIRT3 reportedly up-regulate Mn-SOD expression, though the mechanisms are different for the two sirtuin (i.e., HIF-2α [32] and/or FOXO4 [124] versus FOXO3a [114]). Nuclear localization was required for SIRT1 to up-regulate Mn-SOD [117], and this might also be the case with SIRT3. Sundaresan et al. [115] demonstrated that overexpression of nuclear SIRT3 that lacks the MLS protected cardiomyocytes from genotoxic stress and oxidant stress as did overexpression of wild-type mitochondrial SIRT3. Manganese superoxide dismutase is unlikely to be the only ROS scavenger under regulation by SIRT1. Alcendor et al. [2] recently reported that heart-specific overexpression of SIRT1 inhibited oxidative stress-induced damage by paraquat and that this cardioprotection seemed to be achieved by up-regulation of the protein level of catalase via transcriptional activation of FOXO1a.

It is notable that SIRT3 increases ROS scavenging activity of Mn-SOD by deacetylating K53/K68 [95] and K122 [119] in MEFs, in addition to its effect on protein level of Mn-SOD [114]. SIRT3 also increases activity of other ROS-detoxifying enzymes indirectly. SIRT3 deacetylates and activates the TCA cycle enzyme isocitrate dehydrogenase 2 (IDH2) and glutamate dehydrogenase in murine liver [69, 102], both of which produce NADPH in the mitochondria. NADPH in turn is required for glutathione reductase to convert oxidized glutathione to reduced glutathione, which is a crucial cofactor for mitochondrial glutathione peroxidase to scavenge ROS. Consistent with these findings, Shinmura et al. [107] demonstrated that treatment of cardiomyocytes with resveratrol, an activator of SIRT1 and SIRT3, decreased ROS production and improved cell survival after hypoxia/reoxygenation without increasing the expression level of MnSOD protein.

Recently, mitochondrial aldehyde dehydrogenase 2 (ALDH2) has been identified as a novel target of SIRT3 [72]. Excessive ROS in stressed hearts triggers lipid peroxidation and accumulation of reactive aldehydes, which in turn impairs mitochondrial function and induces cell damage. Aldehyde dehydrogenase 2 reduces the toxicity by removal of the aldehydes, resulting in cardioprotection [23]. Taken together, SIRT3-mediated ALDH2 activation could be another mechanism that mitigates cardiomyocyte damage induced by ROS. However, the protective mechanism may not operate in non-cardiac tissues, as acetaminophen hepatotoxicity was rather exacerbated by ALDH2 in mice [72].

## Regulation of angiogenesis by sirtuins

Cardiac hypertrophy occurs as an adaptive response to increased workload to maintain cardiac function. However, under a prolonged stress condition, this program becomes maladaptive, resulting in myocyte death, fibrosis, ventricular dilation and eventually transition to heart failure. It has been shown that both heart size and cardiac function are angiogenesis dependent, and disruption of coordinated cardiac muscle growth and angiogenesis in the heart contributes to the progression from adaptive cardiac hypertrophy to heart failure [99, 108].

Several lines of evidence indicate that SIRT1 plays crucial roles in compensatory angiogenesis. Potente et al. [94] demonstrated that SIRT1 was highly expressed in the vasculature during blood vessel growth. Knockdown of SIRT1 by siRNA in human umbilical vein endothelial cells (HUVECs) blocked sprouting angiogenesis with down-regulation of genes involved in blood vessel development. Endothelial cell-specific deletion of SIRT1 in mice blunted ischemia-induced neovascularization in the hindlimb [94]. Conversely, activation of SIRT1 by resveratrol induced up-regulation of vascular endothelial growth factor (VEGF) and its tyrosine kinase receptor Flk-1, along with nitric oxide synthase (inducible NOS and endothelial NOS) 3 weeks after myocardial infarction, resulting in increased capillary density in rats [33]. Induction of angiogenesis by SIRT1 seemed to be mediated by inhibition of Foxo1, an essential negative regulator of blood vessel development, because SIRT1 interacts and deacetylates Foxo1 in HUVECs [94].

In addition to direct up-regulation of angiogenic factors, inactivation of p53, an anti-angiogenetic factor, might be a mechanism by which SIRT1 regulates angiogenesis. Sano et al. [99] demonstrated that sustained pressure overload on the left ventricle induces up-regulation of p53. P53 in turn inhibits activity of hypoxia-inducible factor-1 (HIF-1), a transcription factor that regulates the expression of genes involved in hypoxic adaptation including VEGF [47]. Indeed, the inactivation of HIF-1 was causally related to down-regulation of angiogenic factors, reduction in capillary density and transition from adaptive hypertrophy to heart failure [99]. SIRT1 deacetylates and reduces transcriptional activity of p53 in cardiomyocytes [3], and HIF-1 increases the expression level of SIRT1 protein [24]. Thus, fine-tuned balance between activities of SIRT1 and p53 may determine the extent of angiogenesis in the pressure-overloaded heart and transition from compensated hypertrophy to decompensated heart failure. So far, involvement of SIRT3 in angiogenesis has not been reported.

## Modulation of cardiomyocyte Ca^2+^ handling by sirtuins

During excitation–contraction coupling, Ca^2+^ entry through L-type Ca^2+^ channels triggers Ca^2+^ release from the sarcoplasmic reticulum (SR) through ryanodine receptors, raising free intracellular Ca^2+^ concentration from the nanomolar range in the diastole to the micromolar range in the systole. The increase of the concentration allows Ca^2+^ to associate with troponin C, a myofilament protein, resulting in sarcomere shortening and muscle contraction. Subsequent muscle relaxation is initiated by Ca^2+^ dissociation from the troponin complex, when Ca^2+^ is removed from the cytoplasm by Ca^2+^ handling machinery. In humans, SERCA2a is responsible for 70 % of the removal by taking up Ca^2+^ into the SR, and the rest is extruded by the Na^2+^/Ca^2+^ exchanger (28 %) and plasma-membrane Ca^2+^ ATPase (2 %) [40]. Earlier studies demonstrated that intracellular Ca^2+^ handling was abnormal in the failing human myocardium, and this abnormality appears to be one of the mechanisms responsible for systolic and diastolic dysfunction [39]. Particularly, impaired Ca^2+^ uptake into the SR through SERCA2a has been demonstrated to be associated with heart failure; SERCA2a mRNA was decreased in patients with dilated or ischemic cardiomyopathy [6] and aging rat hearts [75], and down-regulation of SERCA2A protein and/or impaired SERCA2a function was observed in animal model of diabetic cardiomyopathy [15, 26] and human dilated cardiomyopathy [67].

Knowledge of the role of sirtuins in intracellular Ca^2+^ regulation in cardiomyocyte is limited. However, a study has shown that reduced SERCA2a protein level, ventricular dysfunction, ventricular dilatation and mortality in a mouse model of type-1 diabetes were nearly normalized by treatment with resveratrol [112]. They also demonstrated that in cultured cardiomyocytes, SERCA2a promoter activity that was highly repressed by high-glucose media was significantly improved by resveratrol in an SIRT1-dependent manner [112]. In light of the universal nature of down-regulation of SERCA2a protein/mRNA and its dysfunction in heart failure, whether activation of SIRT1 ameliorates heart failure irrespective of the etiology warrants investigation.

## Regulation of cell death/survival by sirtuins

Loss of cardiomyocytes and their replacement with reactive fibrosis are important causative factors in the development of heart failure. Cardiomyocyte death in failing hearts is induced by multiple mechanisms: apoptosis, necrosis, and autophagic cell death [58]. SIRT1 and SIRT3 have been demonstrated to modify these types of cell death via diverse mechanisms as described below.

### Modulation of apoptotic machinery

Ku70 has been recognized as a subunit of the Ku protein complex, which plays an important role in DNA damage repair. Ku70 is associated with Bax, a pro-apoptotic Bcl-2 family protein, in its deacetylated form. However, acetylation of Ku70 induced by cellular stress facilitates the release of Bax from Ku70 and induces its mitochondrial translocation, resulting in apoptosis [28, 100]. Caloric restriction up-regulates SIRT1 and maintains residues K539 and K542 of Ku70 in a deacetylated form, keeping Bax sequestered away from mitochondria, thereby inhibiting stress-induced apoptosis in 293 cells [29]. Recently, Nagalingam et al. showed that SIRT3 is catalytically active outside the mitochondria and also targets Ku70. They demonstrated that deacetylation of Ku70 by SIRT3 promotes Ku70/Bax interaction in Hela cells, and this makes cells resistant to Bax-mediated cell damage [115], as was the case with SIRT1-induced Ku70 deacetylation. Full-length SIRT3-expressing cells were resistant to cell death induced by MNNG (*N*-methyl-*N*′-nitro-*N*-nitrosoguanidine) even when SIRT1 was knocked out, indicating that SIRT1 and SIRT3 have redundant functions to protect cells from apoptosis [115].

p53 is the first non-histone substrate found to be deacetylated by SIRT1 [74]. Acetylation of p53 is required for recruitment of transcription co-factors of PUMA and Bax, proapoptotic genes, to their promoter regions and activation of the promoter [21]. SIRT1 deacetylates p53 and silences the pro-apoptotic activity of p53 [110]. SIRT1-deficiency was associated with hyperacetylation of p53 and increased sensitivity to apoptosis induced by ionizing radiation in thymocytes [25]. Inhibition of SIRT1 by nicotinamide (NAM), an SIRT1 inhibitor, elevated the acetylation level and activity of p53, resulting in cardiomyocyte death. Conversely, expression of dominant negative p53 prevented NAM-induced cardiomyocyte death [3]. In failing hearts, the increased activity of poly(ADP-ribose) polymerase-1, which induces NAD^+^ depletion, was associated with reduced SIRT1 activity and increased acetylation of p53 at K373/K382 [90]. Collectively, these findings support the notion that p53 deacetylation by SIRT1 is crucial for cardiomyocyte survival.

It has also been reported that SIRT1 suppressed apoptosis via mechanisms that were independent of the deacetylase activity. The protective effect of SIRT1 in neurons against low potassium-induced apoptosis was observed even after pharmacological inhibition of SIRT1 by nicotinamide and sirtinol, or transfection of mutant SIRT1 that lacks deacetylase activity [89]. These observations indicate that non-catalytic mechanisms may also play a role in SIRT1-mediated cell survival under certain stresses in some types of cells.

SIRT3 was also found to interact with p53. SIRT3 directly deacetylates p53, thereby abrogating its activity to execute growth arrest and senescence in bladder carcinoma cells [65]. However, the role of SIRT3 in regulation of p53-mediated apoptosis in cardiomyocytes has not yet been clarified.

### Suppression of mPTP opening

The mitochondrial permeability transition pore (mPTP) is a large conductance channel in the mitochondrial inner membrane that non-selectively passes molecules <1.5 kDa in response to its opening stimuli. Mitochondrial permeability transition pore opening has been shown to be involved in cell necrosis by a variety of causes including ischemia/reperfusion and anti-cancer agents [48, 129]. While acute robust irreversible opening of the mPTP can lead to cell necrosis, chronic low-level opening of the mPTP induces swelling and membrane depolarization of mitochondria and removal of defective mitochondria by autophagy [56]. Indeed, mitochondria of young animals are relatively small and bioenergetically efficient, but with aging they become swollen and less numerous, and chronically depolarized [127], leading to decreased mitochondrial function, decreased tolerance to stress and increased susceptibility to cell death [19].

Accumulating evidence indicates that sirtuins are involved in the regulation of stimuli for mPTP opening and the mPTP itself. The levels of intracellular ATP and ROS and intramitochondrial Ca^2+^ are major determinants of the threshold of mPTP opening, and these factors are critically regulated by SIRT1 and/or SIRT3 as discussed above. Contribution of p53 to mPTP-mediated necrosis is indicated by the findings that an inhibitor of p53, pifithrin-α, sensitized the myocardium to cardioprotection afforded by isoflurane and that this beneficial effect of pifithrin-α was abolished by atractyloside, an activator of mPTP [125]. The impact of sirtuin-mediated regulation of p53 on mPTP opening remains to be determined.

As for direct modulation of the mPTP by sirtuins, interaction of SIRT3 with cyclophilin D (CyPD) has been recently reported. Cyclophilin D is one of the cyclophilin family proteins with peptidylprolyl isomerase (PPIase) activity and localizes primarily in the mitochondrial matrix under baseline conditions. In response to cellular stress such as ischemia/reperfusion, CyPD interacts with inorganic phosphate carrier and adenine nucleotide translocase on the mitochondrial inner membrane in cardiomyocytes, leading to sensitization of the mPTP to opening stimuli [79]. In fact, genetic ablation of CyPD or treatment with cyclosporine A, an inhibitor of CyPD, elevates the threshold for opening of the mPTP and affords tolerance against ischemia/reperfusion-induced necrosis [9, 10, 27, 81, 93]. Shulga et al. [109] showed that SIRT3 deacetylates CyPD and inhibits its PPIase activity in HeLa cells. Furthermore, Hafner et al. [41] confirmed that CyPD was deacetylated at Lys166 by SIRT3 in cardiac tissue. They also demonstrated that an increase in Ca^2+^ sensitivity of the mPTP by aging was significantly enhanced in cardiac mitochondria isolated from SIRT3-knockout mice. These findings indicate that SIRT3 counteracts increased sensitivity of the mPTP in response to cellular stress in failing and aging hearts.

### Regulation of cell-protective signaling

Sensing intracellular energy balance, AMP-activated protein kinase (AMPK) is activated to reserve cellular energy content. Activated AMPK can promote the metabolic pathways relevant to ATP production such as cellular glucose uptake and fatty acid oxidation, while it switches off ATP-consuming anabolic pathways. AMP-activated protein kinase also serves as a key regulator of cell survival in response to pathological stress, such as ischemia/reperfusion, endoplasmic reticulum stress and hypoxia [46, 87, 120]. SIRT1 and SIRT3 activate serine–threonine liver kinase B1 (LKB1), one of the many upstream kinases of AMPK, in human embryonic kidney 293 cells [61] and in cardiomyocytes [92], respectively. Furthermore, the interplay between sirtuins and AMPK might be reciprocal, because AMPK enhances activity of SIRT1 by enhancing the intracellular NAD^+^/NADH ratio in C2C12 myocytes [22]. Cardioprotective effects afforded by exogenous NAD^+^ were mediated by SIRT3-induced LKB1 activation and subsequent AMPK phosphorylation [92]. There are many downstream targets of AMPK [14], among which inhibitory phosphorylation of GSK3β [92, 105] and up-regulation of GLUT4 protein [87] appear to play a role in cardioprotection.

Nuclear localization of SIRT1 was necessary for up-regulation of Mn-SOD protein and cell protection against necrosis under oxidant stress in C2C12 myoblast cells [117]. In this context, signals that facilitate nuclear import of SIRT1 should be important for SIRT1 to protect cells from death. Nuclear localization of SIRT1 in cardiomyocytes was inhibited by use of the PI3K/Akt inhibitor LY294002, suggesting a role of Akt-mediated phosphorylation in the nuclear translocation of SIRT1 [118]. Similarly, human SIRT1 was phosphorylated by JNK1 on Ser27, Ser47 and Thr530 under oxidative stress, and this phosphorylation of SIRT1 increased its nuclear localization and enzymatic activity. Interestingly, JNK1-induced phosphorylation of SIRT1 showed substrate specificity resulting in deacetylation of histone H3 but not that of p53 [83]. Six-month calorie restriction in rats significantly increased the protein level of SIRT1 in the nucleus, deacetylation of histone H3 and myocardial tolerance against ischemia/reperfusion injury. Treatment with *N*-nitro-l-arginine methyl ester, an inhibitor of nitric oxide (NO) synthase, prevented the increase in nuclear Sirt1 and cardioprotection in calorie-restricted animals [106]. These findings indicate a role of NO in nuclear translocation of SIRT1 (Fig. 2), but relationships of NO with Akt and JNK in the regulation of intracellular localization of SIRT1 remain to be determined.

## Regulation of autophagy by sirtuins

Autophagy is a dynamic process of intracellular bulk degradation in which cytosolic proteins and organelles are sequestered into double-membrane vesicles called autophagosomes to be fused with lysosomes for degradation. In nutrient-deprived cells, autophagy serves as a cell survival mechanism by degrading intracellular proteins and lipids to recycle them for generation of ATP [76, 80]. Under stressed conditions, autophagy selectively removes damaged mitochondria [56]. Since damaged mitochondria release pro-apoptotic factors such as cytochrome c, autophagy can prevent activation of apoptotic machinery [44]. In cardiac tissues, autophagy has been demonstrated to play a protective role [37]. Enhancing autophagy by beclin1 overexpression reduces Bax activation and protects cardiac HL-1 cells against ischemia/reperfusion injury [43]. Tamoxifen-induced temporal and cardiac-specific knockout of Atg5, a protein involved in autophagosome formation, led to left ventricular dilatation and contractile dysfunction with misalignment and aggregation of mitochondria in mice [82]. Akt overexpression in mice suppressed autophagy, which was associated with cardiac hypertrophy, interstitial fibrosis and contractile dysfunction [52].

SIRT1 regulates autophagy by interacting with and deacetylating autophagy-related proteins Atg5, Atg7 and Atg8 [62]. Recently, Hariharan et al. [45] demonstrated that SIRT1 was required for starvation-induced autophagy in cardiomyocytes, in which SIRT1-mediated deacetylation of FOXO1 and subsequent activation of Rab7 play a role. Furthermore, FOXO1 was indispensable for maintenance of cardiac function after starvation. Thus, it is plausible that autophagy induced by activation of the SIRT1-FOXO1 axis is an important adaptive mechanism in the failing heart, which is potentially starved of energy.

## Conclusion

**Fig. 3:**
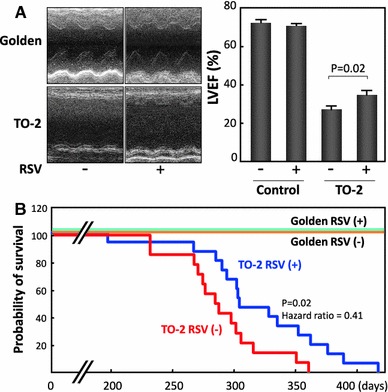
Preserved cardiac function and improved survival by oral administration of resveratrol in TO-2 cardiomyopathic hamsters. Resveratrol was orally administered to control golden hamsters and TO-2 hamsters from the age of 6 weeks. **a** Echocardiography performed at the age of 30 weeks revealed that the administration of resveratrol attenuated the deterioration of cardiac function inTO-2 hamsters (LVEF 34.4 ± 2.2 vs. 27.9 ± 1.6 % in untreated TO-2 hamsters). **b** A Cox proportional hazards regression showed that resveratrol significantly reduced the risk of death inTO-2 hamsters by 59 % (hazard ratio = 0.41, *p* = 0.02). RSV: treatment with resveratrol at 4 g/kg chow, Golden: golden hamsters (control). Produced from ref [117] with permission

Sirtuins are a unique class of proteins that link acetylation status of proteins to a wide variety of physiological functions and diseases. Our understanding of the biology of SIRT1 and SIRT3 has expanded considerably over the past decade, with numerous novel targets being identified. The bulk of emerging evidence indicates that these sirtuins are involved in numerous biological functions including regulation of energy production, detoxification of oxidative stress, promotion of angiogenesis, intracellular Ca^2+^ handling, suppression of cell death and induction of autophagy. Since derangement of these physiological processes underlies the development/progression of heart failure, pharmacologic activation of SIRT1 and/or SIRT3 potentially ameliorates the disease. In agreement with this notion, oral administration of resveratrol preserved cardiac function and improved survival in various animal models of heart failure (Fig. 3). Although more work is needed to fully understand the role of sirtuins in cardiac cell biology, a sirtuin-activating compound could be one of the promising future therapeutic approaches for heart failure.
